# Differential effects of prior versus concomitant Steroid and Antibiotic Treatment on Immunotherapy Efficacy - A Pooled Analysis of the RAMONA, INTEGA, OPTIM, ELDORANDO, FORCE, TITAN-RCC and TITAN-TCC Trials of the German AIO Study Group

**DOI:** 10.1038/s41416-026-03428-8

**Published:** 2026-05-19

**Authors:** Isabella C. Wiest, Lena Dreikhausen, Ralph Keller, Marcos Marin-Galiano, Laurence Albiges, Johannes Meran, Katharina Leucht, Stefan Rieken, Emilio Esteban, Friedemann Zengerling, Joseph Tintelnot, Marc-Oliver Grimm, Farastuk Bozorgmehr, Petros Christopoulos, Alexander Stein, Mascha Binder, Nicolai Härtel, Konrad Klinghammer, Viktor Grünwald, Michael Pogorzelski, Matthias P. Ebert

**Affiliations:** 1 Department of Medicine II, Medical Faculty Mannheim, Heidelberg University, Mannheim, Germany; 2 Else Kroener Fresenius Center for Digital Health, Technical University Dresden, Dresden, Germany; 3 AIO-Studien-gGmbH, Berlin, Germany; 4 M.A.R.C.O. Institute for Clinical Research and Statistics, Düsseldorf, Germany; 5 Gustave Roussy, INSERM, Biothérapies Innovantes U1363, University of Paris Saclay, Villejuif, 94800 France; 6 Department of Internal Medicine, Hematology and Internal Oncology, Hospital Barmherzige Brueder, Vienna, Austria; 7 Department of Urology, Jena University Hospital, Friedrich-Schiller University, Jena, Germany; 8 Comprehensive Cancer Center Germany (CCCG), Jena, Germany; 9 Universitätsklinik & Poliklinik für Strahlentherapie & Radioonkologie, Universitätsmedizin Göttingen (UMG), Göttingen, Germany; 10 Department of Medical Oncology, Hospital Universitario Central de Asturias, Oviedo, Spain; 11 Department of Urology, Ulm University Hospital, Ulm, Germany; 12 Department of Medicine II, University Medical Center Hamburg-Eppendorf, University Hamburg, Hamburg, Germany; 13 Dept. of Thoracic Oncology, Thoraxklinik, Heidelberg University Hospital and National Center for Tumor Diseases (NCT), NCT Heidelberg, a partnership between DKFZ and Heidelberg University Hospital, Heidelberg, Germany; 14 Translational Lung Research Center Heidelberg (TLRC-H), Member of the German Center for Lung Research (DZL), Hamburg, Germany; 15 Hematology-Oncology Practice Eppendorf (HOPE), Hamburg, Germany; 16 University Cancer Center Hamburg, Hamburg, Germany; 17 Medical Oncology, Unispital Basel, Basel, Switzerland; 18 Department Biomedicine, Laboratory Translational Immunooncology, University and University Hospital Basel, Basel, Switzerland; 19 Charite Universitätsmedizin Berlin, Hämatologie Onkologie, Berlin, Germany; 20 University Hospital Essen, Department of Medical Oncology, West German Cancer Center, Essen, Germany; 21 Carolus Institute for Urologic Oncology, West-German Cancer Centre Essen, University Hospital Essen, Essen, Germany; 22 DKFZ Hector Cancer Institute at the University Medical Center, Mannheim, Germany; 23 Molecular Medicine Partnership Unit, EMBL, Heidelberg, Germany

**Keywords:** Cancer immunotherapy, Cancer

## Abstract

**Background:**

We explored the association of immune-related adverse events (irAE), along with prior and concomitant antibiotic and steroid use, on oncological outcomes following immune checkpoint inhibitor (ICI) treatment in various solid tumours.

**Methods:**

Pooled data from seven trials on ICI therapy across multiple cancer types (head and neck, non-small cell lung cancer, gastroesophageal junctional adenocarcinoma, oesophageal, renal cell, and urothelial carcinoma) was analysed, focusing on overall survival (OS) and progression-free survival (PFS) and antibiotic or steroid use before and during the study.

**Results:**

Of 693 patients, 80 used steroids and 52 used antibiotics prior to the study, while 360 and 331, respectively, used them concomitantly. Lack of prior antibiotic use was associated with longer OS (No vs. Yes: HR 0.552, 95%-CI 0.370–0.822, *p* = 0.0035) and PFS (No vs. Yes: HR 0.703, 95%-CI 0.485–1.019, *p* = 0.0625), whereas concomitant antibiotic use had no such effect. Patients with concomitant steroid use demonstrated longer PFS (No vs. Yes: HR 1.359, 95%-CI 1.091–1.693, *p* = 0.0061).

**Discussion:**

Our study confirmed associations between antibiotic and steroid use and ICI efficacy in cancer. Prior, but not concomitant, antibiotic use was linked to reduced OS, supporting the role of microbiome diversity in tumour response. Concomitant steroid use was associated with improved PFS, potentially reflecting its link to irAE occurrence.

## Introduction

Immunotherapy has revolutionized cancer treatment: It activates anti-tumour response by targeting the patient’s immune system. Many different mechanisms altering the patient’s immune response can be addressed. Mainly, inhibitory signals of T-cell activation are downregulated which leverages the cytotoxic function of T-cells against cancer cells. Immune checkpoint inhibitors (ICI) comprise antibodies that block programmed death-1 or programmed death ligand 1 (PD-1/PD-L1) or cytotoxic T lymphocyte antigen 4 (CTLA-4) [1]. These drugs are approved for about 20 cancer entities and showed impressive tumour recurrence in some patients, whereas many patients do not respond to these therapies or acquire resistance at a later time in the therapy course [2].

Intervening in checkpoint signalling harbours a toxicity profile different from that of conventional chemotherapeutic regimens [3]. Immune-related adverse events (irAE) occur in up to 76% of patients receiving ICI. They range from mild to life-threatening events and often affect the liver, the lungs, the skin and the endocrine system [4]. Management of irAE involves high-dose corticosteroids, however, their effect on ICI efficacy remains unclear [1, 5]. Previous studies indicate that timing of corticosteroid use is of importance and that primarily their use prior to ICI and at the time of ICI start is associated with worse outcomes [1, 6, 7]. Patients receiving steroid treatment at baseline tend to have more advanced tumours, distant metastasis, and poorer ECOG performance scores, yet steroids still significantly influenced outcomes even after accounting for these factors in a multivariate analysis [1, 8]. Additionally, gut bacteria influence the response to ICI and antibiotic consumption was linked with poor response to ICI [9–12]. However, the combined impact of steroid and antibiotic use on ICI efficacy, particularly across multiple cancer types, remains insufficiently explored.

This study’s aim is to investigate the impact of steroid and antibiotic use on the efficacy of ICI in cancer treatment in a pooled cohort of seven clinical trials examining ICI therapy across multiple cancer types (head and neck, non-small cell lung cancer, gastroesophageal junctional adenocarcinoma, oesophageal, renal cell and urothelial carcinoma). To control for potential confounders, we performed univariate and multivariate analysis on several parameters such as demographics, tumour characteristics, treatment characteristics, comedications, autoimmune disorders and laboratory markers.

This comprehensive post-hoc analysis of seven clinical studies evaluates the effects of various clinical, tumour- and patient-related parameters in patients with solid cancers receiving ICI therapy.

## Methods

### Study design

This study aimed to evaluate the impact of steroid and antibiotic use on the efficacy of immune checkpoint inhibitors in cancer therapy. The study was conducted as a retrospective post-hoc analysis of seven pooled clinical studies, which tested ICI treatment in patients with different solid tumours.

### Study identification and data sources

Clinical studies on ICI treatment in patients with solid tumours, conducted by AIO-Studien-gGmbH and reporting results, were deemed eligible. The AIO-Studien-gGmbH is a non-profit organization which conducts clinical research within the German Cancer Societies. The following seven academic studies were selected for data pooling (Supplementary Table 1 lists all National Clinical Trial Numbers (NCT) and Full Text Titles of included studies): NCT03193931 - “ELDORANDO”, NCT03044626 - “FORCE”, NCT03409848 - “INTEGA”, NCT03620123 - “OPTIM”, NCT03416244 - “RAMONA”, NCT02917772 - “TITAN RCC”, NCT03219775 - “TITAN TCC”.

### Data pooling and harmonization

Treatments were categorized into four distinct groups based on the involvement of ICI. The ICI mono group included patients treated exclusively with ICI, while the ICI dual group received a dual ICI combination therapy. The ICI other category comprised patients undergoing ICI therapy in combination with other treatments. Lastly, the non-ICI group consisted of those receiving therapy without any ICI component (Supplementary Table 2 provides an overview of the study arms, detailing the drugs used and their classification within the respective treatment groups). For studies with a randomization after a run-in phase, only the phase after randomization was assessed. For the TITAN studies, patients who have received Ipilimumab boosts were counted towards the ICI dual group, while patients without those boosts were grouped within the ICI mono group. Patients were assessed in different subgroups according to a wide variety of baseline and tumour characteristics. Details about medication classification and laboratory value harmonization can be found in the Supplementary materials. Data extraction was managed and statistical analyses were performed using SAS version 9.4.

### Objectives

The two main objectives of the pooled analyses were to describe the occurrence of IrAE (in general and for different irAE subgroups as described in Supplementary Table 3) and to determine predictors for their occurrence. The second main objective was to identify predictors for the efficacy of the ICI treatment. The outcome measures were overall survival (OS), progression-free survival (PFS), duration of response (DOR) and treatment-free survival (TFS) as well as objective response (OR) and disease control rate (DCR). Special focus of this analysis was the impact of steroid and antibiotic use on the efficacy of ICI in cancer treatment.

### Statistical analysis

For the statistical analysis, two populations were defined: The eligible safety set (ES) and the per protocol set (PP). The ES contains all patients who received study medication at least once (all studies except OPTIM and RAMONA). For OPTIM, all patients who received study medication after randomisation were included and for RAMONA, all patients who received study medication after safety assessment were included. The PP set includes all patients from the ES set who also met the per-protocol criteria of their respective study. The toxicity objective was analysed for the ES set, while the efficacy objective was analysed for the ES and PP sets. As the ES is closest to an intention-to-treat set, we reported results for the ES. Descriptive statistics, including mean, standard deviation, median, minimum, maximum, and interquartile range (1st and 3rd quartiles), were calculated for the pooled dataset. Subgroups were built based on treatment type, cancer type, and irAE occurrence. A detailed breakdown of subgroup definitions and grouping criteria is provided in Supplementary Materials: Subgroup Definitions and Grouping Criteria.

We performed multivariate analysis based on the results of the subgroup analysis for toxicity and efficacy objectives. For the toxicity objective, probabilities were modelled with stepwise logistic regression for the probability of at least one irAE, at least one serious irAE, more than one irAE, at least one irAE with CTCAE grade ≧3, and rarest irAE being common, uncommon and rare. For the efficacy objective, we analysed time-related endpoints such as OS, PFS, DOR, and TFS with the Kaplan–Meier method. Multivariate analysis was performed with a stepwise Cox regression. ORR and DCR were analysed by providing absolute and relative frequencies per subgroup. Subgroups were compared with Fisher’s exact test and multivariate logistic regression. Multivariate analysis used stepwise regression to identify predictors of toxicity and efficacy. The full methodological details, including selection criteria for independent variables and handling of collinearity, are provided in Supplementary Materials: Detailed Methodology for Multivariate Analysis and Model Selection.

## Results

### Study overview and patient characteristics

**Fig. 1:**
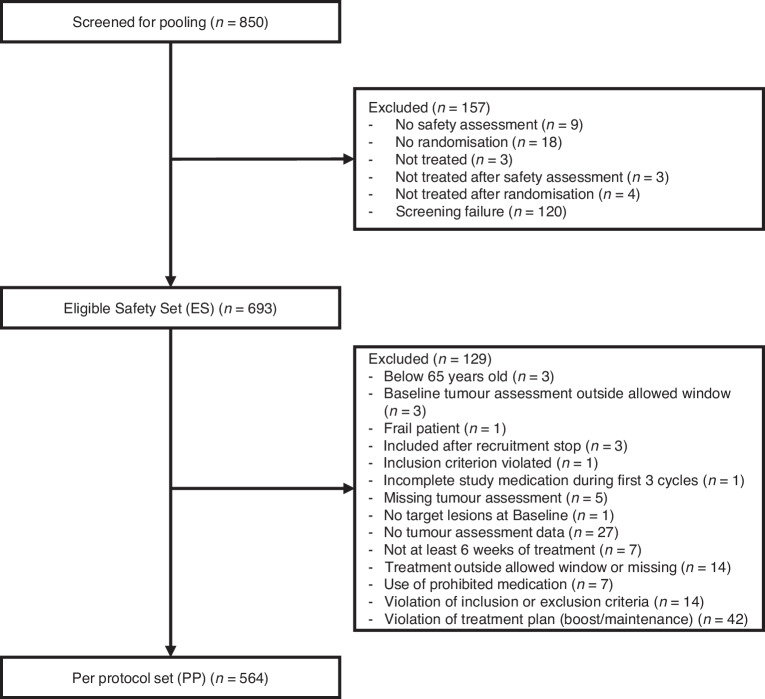
CONSORT - Diagram for eligible safety (ES) and per protocol (PP) set.

The pooling of seven clinical studies included the screening of 850 patients from which 693 patients entered the eligible safety set (ES) and 564 were included in the per protocol set (PP) (Fig. 1 presents a CONSORT diagram illustrating the pooled cohort). Data which was excluded from multivariate analysis was less than 5%. Most of the patients included were patients from the TITAN RCC study (30.0%), followed by the TITAN TCC study (24.4%) and the FORCE study (14.6%). 12.6% of the patients were from the INTEGA study, 7.8% from RAMONA, 6.5% from ELDORANDO and only 4.2% from the OPTIM study. Most of the patients had non-squamous cell carcinoma (Non-SCC) (*n* = 565) and the minority suffered from squamous cell carcinoma (SCC) (*n* = 128). 346 patients received a combination of two ICI (ICI dual), 225 received only one ICI (ICI mono). 83 patients had other therapeutic regimens combined with any kind of ICI (ICI other) and 39 patients received a therapy without an ICI component (Non-ICI) (Table 1 presents an overview of patients by study within the eligible safety and per-protocol set). The pooled patient population had a median age of 66 years, with 70.1% male and 29.9% female patients. Eastern cooperative oncology performance status (ECOG) at baseline was 0 for 55.0%, 1 for 38.7% and 2 for 6.3% of the patients. 40.1% of all patients received chemotherapy as prior cancer treatment, 5.1% received radiotherapy, 24.8% received both, 29.6% received neither and for 0.4% this information was not given. Patient characteristics for all patients are displayed in Table 2.

**Table 1:** Overview of patients by study in eligible safety and per protocol set

	ELDORANDO	FORCE	INTEGA	OPTIM	RAMONA	TITAN RCC	TITAN TCC
Screened	54	110	97	54	75	258	202
Eligible Safety	45	101	87	29	54	208	169
Per Protocol	35	77	79	26	49	160	138
Type of Cancer							
SCC	45	0	0	29	54	0	0
Non-SCC	0	101	87	0	0	208	169
Type of treatment							
ICI mono	22	61	0	0	10	68	64
ICI dual	0	0	44	13	44	140	105
ICI other	0	40	43	0	0	0	0
Non-ICI	23	0	0	16	0	0	0

The eligible safety set comprises all patients who received study medication at least once, except for OPTIM and RAMONA. From OPTIM, all patients who received study medication after randomization were included. From RAMONA, all patients who received study medication after safety assessment were included. The Per Protocol Set consists of all patients who are in the eligible safety set and in the per protocol set of the respective study, which means that it includes those patients who completed the treatment originally allocated.

*SCC* Squamous Cell Carcinoma, *ICI* Immune-Checkpoint Inhibitors.

**Table 2:** Summary statistics for patient characteristics

Variable	*N*	Mean	SD	Minimum	Q1	Median	Q3	Maximum
Age at Baseline [years]	693	65.7	10.22	20	59	66	73	90
Weight at Baseline [kg]	691	76.19	16.613	38	64.3	75	85.7	134
Height at Baseline [cm]	691	172.1	9.15	142	166	173	178	199
Systolic BP at Baseline [mmHg]	690	129.3	18.91	75	118	130	141	217
Diastolic BP at Baseline [mmHg]	690	76.8	11.26	45	70	77	84	116
Heart rate at Baseline [bpm]	686	78.8	13.62	42	69	77	87	135
Body temperature at Baseline [celsius]	686	36.48	0.506	35	36.2	36.5	36.8	38.4
BMI at Baseline [kg/m2]	690	25.62	4.769	13.6	22.24	25.00	28.34	45.2
Tumour proportion score	625	8.4	20.93	0	0	0.5	3	100
Combined positive score	167	9.9	17.81	0	0	3	10	101

*BP* Blood Pressure, *BMI* Body Mass Index.

### Dual ICI therapy is associated with increased toxicity

**Fig. 2:**
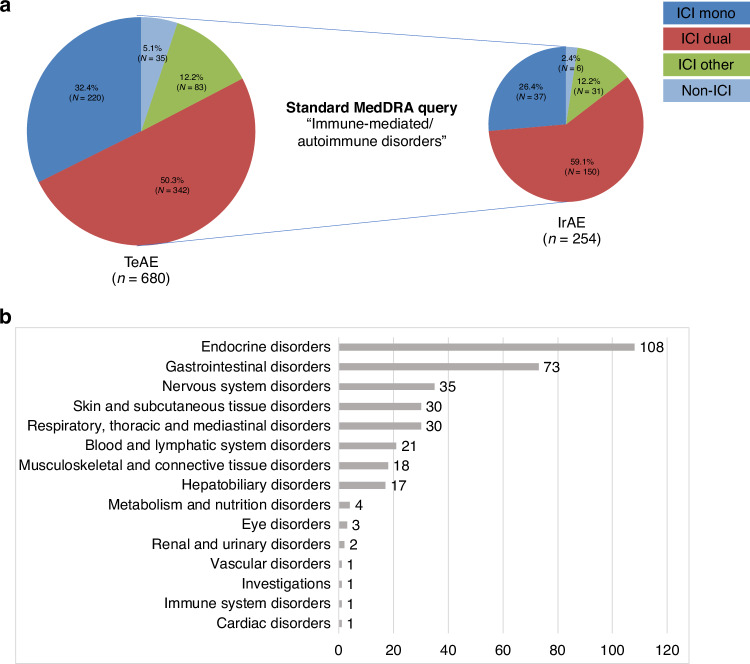
Frequencies of Treatment emergent and immune related adverse events. **a** All Treatment emergent adverse events (TeAE) were filtered with a standard MedDRA query for “immune-mediated and autoimmune disorders” in order to determine immune related adverse events (IrAE). **b** depicts the frequencies of the different system organ classes affected by irAE. ICI: Immune checkpoint inhibitor, MedDRA: Medical Dictionary for Regulatory Activities, irAE: immune related Adverse Events, TeAE: Treatment Emergent Adverse Event.

685 (98.8%) patients experienced at least one adverse event (AE), 680 patients (98.1%) experienced treatment emergent adverse events (TEAE). 73.7% experienced at least one TEAE with CTCAE grade 3 or more (CTCAE grade 1: 85.6%, grade 2: 81.0%, grade 3: 64.1%, grade 4: 14.9%, grade 5: 22.2%, unknown grade: 5.2%). 254 (36.7%) patients had TEAE classified as irAE of whom 90 (13.0%) patients had at least one irAE CTCAE grade 3 or more. Patients receiving dual ICI therapy experienced more irAE than those with ICI monotherapy (Estimate of difference: 13.6%, *p* = 0.0011). Most prevalent irAE were endocrine and gastrointestinal disorders (Fig. 2B).

### Concomitant Steroid and Antibiotic Use was more frequent among patients who experienced irAEs or severe irAEs

No significant difference in irAE occurrence was seen in patients receiving steroids or antibiotics prior to trial start compared to those that did not receive this medication. IrAE occurrence was significantly higher in the group of patients with concomitant steroid use (Estimate of difference (EoD): 21.4%, *p* < 0.0001). Additionally, serious irAE (EoD: 15.8%, *p* < 0.0001) and multiple irAE (EoD: 19.7%, *p* = 0.0028) as well as the occurrence of irAE with CTCAE grade 3 or more (EoD: 25%, *p* < 0.0001) and rare irAE (EoD:11.9%, *p* = 0.0404) were more frequent in this group. Patients who received antibiotics concomitantly had significantly more irAE (EoD 14.3%, *p* = 0.0001) in general as well as more serious irAE (EoD 8.1%, *p* = 0.0017) and rare irAE (EoD 13.0%, *p* = 0.0164) in multivariate analysis (ES). These patients showed a trend towards multiple irAE (EoD 11.9%, *p* = 0.0698), and irAE with CTCAE grade 3 or more (EoD: 11.7%, *p* = 0.0634).

In multivariate logistic regression analysis for probability of irAE occurrence, all variables examined were included. The most relevant variables influencing the odds of irAE occurrence were concomitant steroids use (*p* = 0.0005), treatment duration (*p* = 0.0008), actual treatment (*p* = 0.0061), concomitant antibiotics (*p* = 0.0018), pre-therapy (*p* = 0.0230), neutrophil count at baseline (*p* = 0.0449), BMI (*p* = 0.0390)(Supplementary Table 4 and Supplementary Table 5). For the occurrence of serious irAE, concomitant steroids (*p* < 0.0001), actual treatment (*p* = 0.0002), cancer type (*p* = 0.0140), concomitant antibiotics (*p* = 0.0305) and liver metastasis at baseline (*p* = 0.0489) were relevant variables in logistic regression (Supplementary Table 6 and Supplementary Table 7).

### Concomitant steroids associated with improved, prior antibiotics with reduced survival

**Fig. 3:**
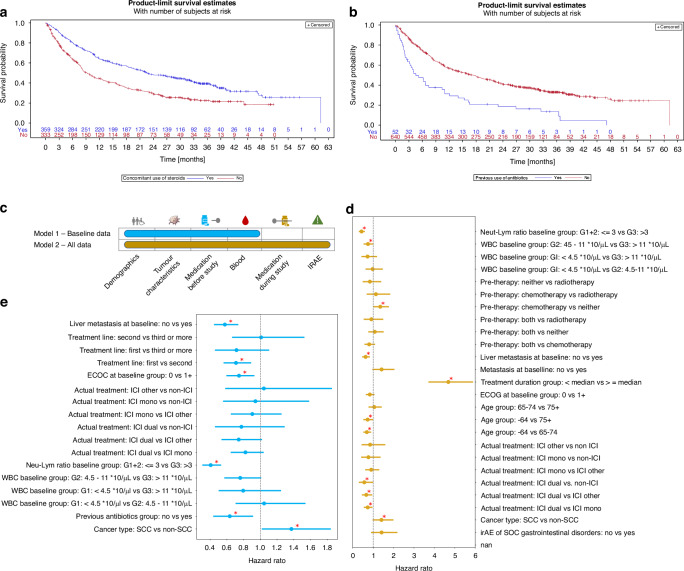
Kaplan–Meier plots for survival. **a** depicts the overall survival curves for patients who received steroids during study treatment (Yes, in black) vs. those who did not (No, in red). **b** shows the plot depicting the survival for patients who received antibiotics prior to study treatment (Yes, black) and those who did not (No, red). **c** shows the variable categories included in model 1 with baseline data and model 2 with all data. **d** Cox regression for analysis of overall survival. The grouped variable categories included in the stepwise model building for Model 2, **e** shows hazard ratios with confidence intervals from variables included in the stepwise modelling process for Model 1. Cox proportional hazards regression was applied. Model building used stepwise selection, with variables entered or removed at *p* = 0.20 based on Chi-square tests. For the final model, the Chi-square *p*-value is reported, and hazard ratios are presented with Wald confidence limits. ECOG: Eastern Cooperative Oncology Group Performance Status, SCC: Squamous Cell Carcinoma, irAE: immune related Adverse Events, WBC: White Blood Cell Count. SOC: System Organ Class, G: Group. *Icon source: Flaticon.com*.

Overall survival (OS) did not differ by the patient groups who received steroid treatment prior to therapy (HR Yes vs. No 1.026, *p* = 0.8590). Patients who received steroids concomitantly to study treatment showed increased OS compared to those who did not receive steroids concomitantly (Median OS concomitant steroids in months: 22.57 (95% CL: 18.87; 29.2) vs. no concomitant steroids: 9.2 (95% CL: 7.9; 12.1), *p* < 0.0001) (Fig. 3a shows the Kaplan–Meier plot). Patients who had antibiotic treatment prior to study treatment showed shorter OS than patients without prior antibiotic treatment (Median OS prior antibiotics in months: 4,57 (95% CL: 2.7 vs. 10.4) vs. 17.43 (95% CL: 13.87; 20.2), *p* < 0.0001) (Fig. 3B). Concomitant antibiotic treatment did not show a survival difference.

Multivariate cox regression for all data (Fig. 3d) revealed longer treatment duration (*p* < 0.0001), higher neutrophil-lymphocyte ratio (NLR) at baseline (*p* < 0.0001), liver metastasis present at baseline (*p* = 0.0002), SCC cancer type (*p* = 0.0453), dual ICI therapy (*p* = 0.0076) and older age (above 65, *p* = 0.0050) as the most influential factors for worse OS (Table 3). Steroid treatment, either prior or during study treatment, had no effect in multivariate analysis. When modelling influencing baseline factors only (Fig. 3E), previous antibiotic treatment had a significant negative effect on OS (*p* = 0.0121) in the multivariate Cox regression, along with high NLR at baseline (*p* < 0.0001), liver metastasis at baseline (*p* < 0.0001), higher ECOG at baseline (*p* = 0.0065), higher treatment line (*p* = 0.0080) and SCC as cancer type (*p* = 0.0337) (Table 3).

**Table 3:** Cox regression for analysis of overall survival (Eligible Safety set)

Subgroup	Overall *p*-value	Comparison	Hazard ratio	Lower 95%-CL	Upper 95%-CL	
Model 1 Baseline Data						
Cancer Type	0.0337	SCC vs non-SCC	1.371	1.025	1.834	Different from HR = 1
Previous Antibiotics Group	0.0121	No vs Yes	0.635	0.445	0.906	Different from HR = 1
WBC Baseline Group	0.1518	G1: < 4.5 *10/µL vs G2: 4.5 - 11 *10/µL	1.045	0.713	1.532	
		G1: < 4.5 *10/µL vs G3: > 11 *10/µL	0.795	0.510	1.240	
		G2: 4.5 - 11 *10/µL vs G3: > 11 *10/µL	0.760	0.577	1.003	
Neut-Lym Ratio Baseline Group	<.0001	G1 + 2: <= 3 vs G3: >3	0.407	0.315	0.525	Different from HR = 1
Actual Treatment	0.1531	ICI dual vs ICI mono	0.822	0.654	1.033	
		ICI dual vs ICI other	0.742	0.544	1.013	
		ICI dual vs Non-ICI	0.774	0.466	1.283	
		ICI mono vs ICI other	0.904	0.655	1.247	
		ICI mono vs Non-ICI	0.941	0.563	1.576	
Model 2 All Data						
At Least One irAE						Not estimable
irAE of SOC Gastrointestinal Disorders	0.1086	No vs Yes	1.414	0.926	2.158	
Cancer Type	0.0453	SCC vs non-SCC	1.405	1.007	1.960	Different from HR = 1
Actual Treatment	0.0076	ICI dual vs ICI mono	0.734	0.578	0.932	Different from HR = 1
		ICI dual vs ICI other	0.663	0.477	0.921	Different from HR = 1
		ICI dual vs Non-ICI	0.565	0.329	0.969	Different from HR = 1
		ICI mono vs ICI other	0.903	0.643	1.268	
		ICI mono vs Non-ICI	0.769	0.444	1.333	
		ICI other vs Non-ICI	0.852	0.464	1.564	
Age Group	0.0050	-64 vs 65-74	0.692	0.550	0.871	Different from HR = 1
		-64 vs 75+	0.731	0.551	0.969	Different from HR = 1
		65-74 vs 75+	1.056	0.803	1.389	
ECOG at Baseline Group	0.1036	0 vs 1+	0.836	0.674	1.037	
Treatment Duration Group	<.0001	< median vs >= median	4.685	3.742	5.866	Different from HR = 1
Metastasis at Baseline	0.0539	No vs Yes	1.413	0.994	2.009	
Liver Metastasis at Baseline	0.0002	No vs Yes	0.630	0.493	0.806	Different from HR = 1
		Both vs Radiotherapy	0.911	0.567	1.462	
		Chemotherapy vs Neither	1.349	1.046	1.741	Different from HR = 1
		Chemotherapy vs Radiotherapy	1.135	0.713	1.807	
		Neither vs Radiotherapy	0.841	0.519	1.363	
WBC Baseline Group	0.1195	G1: < 4.5 *10/µL vs G2: 4.5 - 11 *10/µL	0.973	0.658	1.439	
		G1: < 4.5 *10/µL vs G3: > 11 *10/µL	0.726	0.457	1.156	

The *p*-value of the Chi-Square-test is given. For Hazard ratios, Wald confidence limits (CL) are given.

*ICI* Immune checkpoint inhibitor, *ECOG* Eastern Cooperative Oncology Group Performance Status, *SCC* Squamous Cell Carcinoma, *irAE* immune related Adverse Events, *WBC* White Blood Cell Count, *SOC* System Organ Class, *G* Group.

### Adjusting for immortal time bias revealed modest adverse association

To account for immortal time bias, we calculated a time-dependent Cox Model. The results show that initiation of systemic steroids during ICI treatment was associated with a modest, non-significant increase in the hazard of death (HR 1.21, 95% CI 0.98–1.50). Adjustment for cancer type showed that non-SCC histology remained a strong independent predictor of improved survival (HR 0.46, 95% CI 0.35–0.59). Landmark analyses yielded consistent results. Among patients alive at day 30, steroid exposure before the landmark was not significantly associated with subsequent mortality (HR 1.13, 95% CI 0.85–1.50). In contrast, by the 60-day landmark, steroid use within the first 60 days of therapy showed a statistically significant association with worse survival (HR 1.45, 95% CI 1.13–1.87). Together, these findings suggest that while very early steroid use does not clearly influence outcomes, steroid initiation within the first two months of ICI therapy is associated with a measurable adverse impact on overall survival.

### Concomitant steroids show improved, prior antibiotics reduced PFS

PFS did not differ in the group of patients receiving prior steroid treatment (HR Yes v. No: 1.128, *p* = 0.3599). Concomitant use of steroids was significantly associated with longer PFS (Median PFS concomitant steroids in months: 8.2, 95% CL: 6.6; 10.47, and without 2.83, 95% CL: 2.53; 3.5, *p* < 0.0001), whereas concomitant antibiotic use showed a non-significant trend to a better PFS (Median PFS concomitant antibiotics in months: 6.07 (95% CL:4.63; 7.7) vs. 4.67 (95% CL:3.57; 5.6), *p* = 0.0792). Prior antibiotic use was associated with lower PFS (median PFS in months: 2.15 (95% CL: 1.83; 4.4) vs. 5.57 (95% CL: 4.73; 6.6), *p* < 0.0001).

Multivariate Cox regression analysis for baseline data (Supplementary Fig. 1B) revealed lower NLR, treatment type and earlier treatment line, no liver metastasis at baseline, lower ECOG, absence of previous antibiotic use, and non-SCC cancer type as significant independent indicators for better PFS (Supplementary Table 8). In the multivariate Cox regression analysis for all data (Supplementary Fig. 1C), longer treatment duration, no liver metastasis at baseline, presence of at least one serious irAE, gastrointestinal irAE (trend), lower WBC at baseline, lower NLR, second line treatment vs. third line treatment, presence of concomitant steroids, and less pre-therapy were significant indicators for better PFS. Concomitant use of steroids had a significant effect on PFS (No vs. Yes: HR 1.281, *p* = 0.0170) (Supplementary Table 8).

### Association of steroid and antibiotic use with ORR, TFS, DOR, and DCR

Antibiotic treatment prior to study treatment resulted in shorter DOR (5.98 months vs. 30 months, *p* < 0.0001). Patients who received steroids concomitantly presented a longer duration of response (33.5 months vs. 12.8 months, *p* = 0.0273) (Kaplan–Meier plots are depicted in Supplementary Fig. 2). Prior steroid treatment or concomitant antibiotic use did not show differences in duration of response (Prior steroid treatment: 16.37 months vs. 33.3 months, *p* = 0.1481, Concomitant antibiotic use: 18.33 months vs. 30 months, *p* = 0.5856). The multivariate Cox regression from baseline data revealed previous use of antibiotics (*p* < 0.0001), actual treatment type (*p* = 0.0004) and LDH at baseline (*p* = 0.0377) as well as metastasis at baseline (*p* = 0.0290), the most influential factors for the analysis of duration of response (Supplementary Table 9). The multivariate Cox regression including all data showed that patients had significantly longer duration of response when they received ICI monotherapy (*p* = 0.0001), had longer treatment duration (*p* = 0.0001), did receive concomitant steroids (*p* = 0.0128) or did not receive previous antibiotics (*p* = 0.0031), had no liver metastasis at baseline (*p* = 0.0098) and had low baseline LDH levels (*p* = 0.0174). (Supplementary Table 10).

TFS was longer in patients who had received steroids during study treatment (Median time to event 3.07 months vs. 1.13 months, *p* < 0.0001) and shorter for patients who had received antibiotics prior to study treatment (Median time to event 1.73 months vs. 2.5 months, *p* = 0.0044) (Kaplan–Meier plots are depicted in Supplementary Fig. 3). In multivariate Cox regression for baseline data, patients with low baseline NLR (*p* = 0.0009), higher lymphocyte count at baseline (*p* = 0.0367) treated in a first line setting (*p* = 0.0041), without liver metastasis (*p* < 0.0001) at baseline experienced longer TFS (Supplementary Table 11). Including all data, longer treatment duration (*p* < 0.0001), absence of liver metastasis (*p* < 0.0001) but presence of overall metastasis (*p* = 0.0050) at baseline, lower NLR at baseline (*p* = 0.0003) were associated with longer TFS (Supplementary Table 12).

The group of patients with concomitant steroid use who were responders (ORR) (*n* = 149 of 360 patients, 41.4%) was significantly larger (*p* < 0.0001) than the group of patients without steroid use who responded to treatment (*n* = 75 of 333 patients, 22.5%). 19.2% of all patients with antibiotic treatment prior to study treatment were responders (*n* = 10/52) and 33.4% of patients without prior antibiotic treatment were responders (*n* = 214/641) which presented a significant difference (*p* = 0.0441). 39.9% of all patients with concomitant antibiotic treatment (*n* = 132/331) responded to study treatment whereas only 25.4% (*n* = 92/362) patients without concomitant antibiotics were responders (*p* < 0.0001). However, prior use of steroids or antibiotics did not present as influential predictors in multivariate logistic regression, whereas concomitant antibiotic use was associated with higher odds of overall response (Supplementary Tables 13 and 14).

DCR, defined as all RECIST results being stable disease or better, was higher for patients who received steroids during study treatment (*n* = 65/360 patients, 18.1% vs. *n* = 37/333 patients, 11.1%, *p* = 0.0101). Patients who had received antibiotic treatment prior to study treatment had lower DCR (*n* = 1/52 patients, 1.9% vs. *n* = 101/641, 15.8%, *p* = 0.0036). Previous steroid treatment or concomitant antibiotic treatment had no such effect (All influential factors for DCR are given in Supplementary Tables 15 and 16).

In summary, this pooled analysis analyses several therapy-, tumour- and patient-related factors and suggests that prior antibiotic use is associated with decreased efficacy in patients treated with ICI, whereas concomitant steroid use was associated with increased efficacy.

## Discussion

This pooled analysis identified distinct associations between the timing of antibiotic and steroid exposure and ICI outcomes from data of seven pooled clinical trials. Antibiotic use prior to study treatment was associated with worse PFS and DOR, whereas concomitant antibiotic use had no such effect, suggesting a critical exposure window before ICI initiation and indicating no detrimental effect during ICI treatment. Apparent survival benefits among patients receiving steroids during ICI therapy were attributable to immortal-time bias. Although several baseline factors and concomitant medications correlated with the occurrence of irAEs, these associations may reflect underlying immune activation rather than causal relationships. Mechanistically, irAEs are widely considered markers of effective immune engagement, and patients who develop irAEs often experience superior treatment outcomes [1]. Because steroids are typically administered to manage irAEs, their apparent association with improved survival in unadjusted analyses likely reflects this underlying immune activation rather than a therapeutic benefit of steroids themselves. Consistent with this interpretation, steroid use did not emerge as an independent predictor of OS or PFS in our multivariable models.

Our findings about the detrimental effect of prior antibiotics align with meta-analyses by Huang et al. [13] and Lurienne et al. [14], which reported reduced ICI efficacy in antibiotic-exposed patients, particularly within 60 days before treatment [13–16]. While the mechanisms remain unclear, evidence suggests that an intact microbiome plays a key role in ICI efficacy [17, 18]. Other findings, however, suggest that this is confounded by a sicker patient population which suffers from severe infections, rather than a specific drug interaction that disables the immune system [19, 20]. However, a limitation of this study is the lack of granular data on steroid dosing, which precluded stratification by low, intermediate, or high-dose corticosteroid exposure and corresponding subgroup survival analyses. The absence of any observable effect of concomitant antibiotics in our analysis is consistent with the hypothesis that the critical period of vulnerability precedes ICI initiation, rather than coinciding with ongoing therapy.

Steroids are effective in treating irAE, but their immunosuppressive effect on treatment efficacy is unclear and literature suggests highly different effects depending on timing and duration of administration [1]. Research indicates that higher doses of steroids at baseline may deteriorate ICI anti-tumour efficacy [1, 6, 21]. Steroid initiation more than two months after ICI start has been associated with improved OS, PFS and ORR compared to patients who started steroids within the first two months of ICI therapy [7]. Our time-dependent modelling indicates that the apparent survival advantage of concomitant steroid use in unadjusted analyses is primarily driven by immortal-time bias rather than a protective pharmacologic effect. Once this bias was controlled for, steroid initiation was associated only with a small, non-significant adverse trend, suggesting no strong independent treatment effect. This interpretation is consistent with prior studies in which worse outcomes among steroid-exposed patients were largely attributable to higher disease burden, poorer ECOG performance status, and other adverse prognostic factors rather than to steroid therapy itself [1, 22, 23]. Additional complexities reported in the literature, such as dose dependence [24], systemic versus topical administration, ICI and chemotherapy combinations, and steroid-induced shifts in NLR [1, 25] could not be fully evaluated in our pooled dataset but may contribute to the heterogeneity of reported effects.

The retrospective nature of the pooled dataset introduced expected limitations, including heterogeneous cancer types and treatment settings, incomplete medication dates requiring conservative “worst-case” imputation, and potential exposure misclassification, despite the high proportion of complete records. Restricting prior antibiotic exposure to a ± 30-day window risks misclassifying patients with earlier dysbiosis. Immune-related adverse events were identified using standardized MedDRA query (SMQ) filters applied to all treatment-emergent adverse events, without clinical adjudication. This approach may have led to both over- and under-capture of true irAEs and may blur causal relationships between exposures, mediators, and outcomes, such as antibiotics administered for steroid-treated irAE complications.

Despite these limitations, the dataset’s breadth enabled robust multivariable modelling across diverse tumour types. By leveraging existing clinical studies beyond their original scope, we demonstrate the value of pooled analyses in identifying critical determinants of ICI efficacy and toxicity, while also addressing the limitations of smaller study cohorts. While retrospective pooling cannot replace prospective clinical trials, our findings highlight the potential of real-world data analyses to enhance and validate insights from individual studies. Investing in such approaches can strengthen data foundations for future research and improve the understanding of ICI therapy across diverse clinical settings.

These limitations highlight the need for further data collection, ideally through prospective studies, to validate our findings and gain a deeper understanding of the role of steroids and antibiotics in ICI therapy. Overall, our findings highlight that real-world pooled datasets can reveal clinically relevant associations that warrant prospective validation.

## Supplementary information

Supplement

## Data Availability

The pooled analysis was performed under the lead of AIO Studien gGmbH and is not available publicly.

## Code availability

The statistical analysis code used for all univariate and multivariate analyses was developed and executed by the M.A.R.C.O. Institute for Clinical Research and Statistics (Düsseldorf, Germany). The code is maintained at the Institute, and their code availability and data access policies apply.
