# Supplementary material for: Differential effects of prior versus concomitant Steroid and Antibiotic Treatment on Immunotherapy Efficacy - A Pooled Analysis of the RAMONA, INTEGA, OPTIM, ELDORANDO, FORCE, TITAN-RCC and TITAN-TCC Trials of the German AIO Study Group
Source: Br J Cancer. 2026 May 19;135(4):612–20. doi: 10.1038/s41416-026-03428-8 (PMC13427731; doi:10.1038/s41416-026-03428-8)
Supplement: Supplementary file 1 — Supplement [file 41416_2026_3428_MOESM1_ESM.docx]

# Supplementary Material

## Supplementary Methods

**Data pooling and harmonization**For laboratory values, the different units presented in the study were harmonized and unequivocal erroneous units corrected. Laboratory values were grouped according to limits of normal into low, normal, and high at Baseline. If groups would be populated with less than 10 patients, they were combined. Since medication was not initially coded, a medical expert manually classified it based on a list of all available verbatim terms, without considering the dose. Previous use was defined as all medication prior (30 days) to first study treatment, concomitant use has been defined as medication within 30 days from the beginning of study treatment. In case of incomplete and ambiguous dates, the worst case has been applied. Other missing data has not been imputed. If data was not available for a certain subgroup, that patient was not considered for univariate or multivariate analysis. Immune related adverse events (irAE) were not specifically documented in the original studies. We therefore classified all treatment emergent adverse events (TEAE) as irAE, if selected by a standardized MedDRA query (SMQ).[[28]](https://paperpile.com/c/dENVde/qkYtR)

**Subgroup Definitions and Grouping Criteria**
Summary statistics for demographics, such as age, weight, height at Baseline, and Body Mass Index (BMI) at Baseline were provided for the pooled cohort as well as according to the treatment group. Subgroups of patients were built according to irAE occurrence, treatment and cancer type as well as according to different features (All subgroups built are presented in Supplementary **Table 3,** grouping for type of treatment is specifically explained in **Supplementary Table 2**).

**Detailed Methodology for Multivariate Analysis and Model Selection**Multivariate analysis was performed with a stepwise Cox regression or stepwise logistic regression. Subgroups with a p-value of less than 0.1 in the univariate analysis were added as independent variables for the multivariate analysis. During the model selection process, independent variables entered and left the model with a p-value of 0.2. In instances of clear collinearity between variables, the variable with the lowest p-value of those variables was used.

## Supplementary Tables

**Supplementary Table 1 - National Clinical Trial Numbers and Full Text Titles of included studies.**

| NCT number | Title | AIO study number |
| --- | --- | --- |
| NCT03193931 | A randomized phase II study comparing pembrolizumab with methotrexate in elderly, frail or cisplatin-ineligible patients with head and neck cancers (ELDORANDO) | AIO-KHT-0115 |
| NCT03044626 | Fostering efficacy of anti – PD-1 – treatment: Nivolumab plus radiotherapy in advanced NSCLC (FORCE) | AIO-YMO/TRK-0415 |
| NCT03409848 | Ipilimumab or FOLFOX in combination with Nivolumab and Trastuzumab previously untreated HER2 positive locally advanced or metastastic EsophagoGastric Adenocarcinoma (INTEGA) | AIO-STO-0217 |
| NCT03620123 | A randomized phase II study on the OPTimization of IMmunotherapy in squamous carcinoma of the head and neck(OPTIM) | AIO-KHT-0117 |
| NCT03416244 | A multicenter open-label phase II trial to evaluate Nivolumab and Ipilimumab for 2nd line therapy in elderly patients with advanced esophageal squamous cell cancer (RAMONA) | AIO-STO-0117 |
| NCT02917772 | A phase II single arm clinical trial of a Tailored ImmunoTherapy Approach with Nivolumab in subjects with metastatic or advanced Renal Cell Carcinoma  (TITAN RCC) | 0216-ASG |
| NCT03219775 | A phase II single arm clinical trial of a Tailored ImmunoTherapy Approach with Nivolumab in subjects with metastatic or advanced Transitional Cell Carcinoma (TITAN TCC) | 0416-ASG |

**Supplementary Table 2 - Mapping of treatment arms to treatment group.**

ICI: Immune checkpoint inhibitor

| Study Arm | Study Drug | Treatment Group |
| --- | --- | --- |
| ELDORANDO | | |
| Arm A | Pembrolizumab | ICI mono |
| Arm B | Methotrexate | Non-ICI |
| FORCE | | |
| Group A | Nivolumab+Radiotherapy | ICI other |
| Group B | Nivolumab | ICI mono |
| INTEGA | | |
| Arm A | Trastuzumab+Nivolumab+Ipilimumab | ICI dual |
| Arm B | Trastuzumab+Nivolumab+FOLFOX | ICI other |
| OPTIM | | |
| Arm A | Nivolumab+Ipilimumab | ICI dual |
| Arm B | Docetaxel | Non-ICI |
| RAMONA | | |
| Arm A | Nivolumab+Ipilimumab | ICI dual |
| Arm B | Nivolumab | ICI mono |
| TITAN RCC and TITAN TCC | | |
| Arm A | Nivolumab | ICI mono |
| Arm B | Nivolumab+Ipilimumab | ICI dual |

**Supplementary Table 3 - Subgroups defined and analyzed for the pooled cohort.**

irAE: immune-related adverse events, CTCAE: Common Terminology Criteria for Adverse Events, ICI: Immune checkpoint inhibitor, SCC: Squamous Cell Carcinoma

| **Subgroup** | **Definition** |
| --- | --- |
| irAE | At least one irAE: yes or no  At least one serious irAE: yes or no |
| Patients with at least one irAE | Number of IRAEs: Exactly one IRAE / more than one IRAE  CTCAE grade: No IRAE of CTCAE grade of 3 or higher / At least one IRAE of CTCAE grade of 3 or higher  Rarity of rarest IRAE in a patient: Rare (<1%), Uncommon (1%-10%), Common (>10%). The rarity was determined by PT based on the frequency of such an AE within the pooled data. |
| Type of Tumor | All patients from ELDORANDO, OPTIM, and RAMONA studies are in the squamous cell carcinoma (SCC) group  All patients in the FORCE, INTEGA, TITAN RCC, and TITAN TCC studies are in the non-squamous cell carcinoma (non-SCC) group. |
| Type of Treatment | ICI mono: Treated only with ICI ICI dual: Dual ICI combination therapy ICI other: ICI therapy in combination with other therapy Non-ICI: Therapy without an ICI component |
| Age at enrollment | Three groups: ≦64 years, 65-74 years, ≧75 years |
| Gender | Male, Female |
| BMI at Baseline | Three groups: <25, 25-30, >30 |
| ECOG at Baseline | Two groups: 0, ≧1 |
| Treatment duration | Two groups: <Median, ≧Median (Medians were determined separately for each type of treatment) |
| TPS score | <1%, ≧1% |
| CPS score | <1%, ≧1% |
| Treatment line | 1st, 2nd, 3rd or later  Note: For studies with a run-in phase (e.g. OPTIM), the run-in phase was counted as a treatment line provided that the study treatment is different from the treatment in the run-in phase. |
| Metastasis at baseline | Yes, No |
| Liver metastasis at baseline | Yes, No |
| Type of pre-therapy | None, Chemotherapy, Radiotherapy, Both |
| Use of previous/concomitant steroid treatment | Yes, No |
| Use of previous/concomitant antibiotic treatment | Yes, No |
| Pre-existing auto-immune disease | Yes, No |
| Biomarkers | |
| Albumin | < 3.5 g, L , 3.5 - 5.4 g/L , > 5.4 g/L |
| LDH | < 60 U/L , 60 - 160 U/L , > 160 U/L |
| Lymphocytes | < 0.77 * 10^3/µL , 0.77 - 4.5 * 10^3/µL , > 4.5 * 10^3/µL |
| Leukocytes | < 4.5 * 10^3/µL , 4.5 - 11 * 10^3/µL , > 11 * 10^3/µL |
| Neutrophils | < 2.6* 10^3/µL , 2.6 - 8.5 * 10^3/µL , > 8.5 * 10^3/µL |
| Neutrophils-Lymphocyte Ratio | < 0.7 , 0.7 – 3 , > 3 |
| Eosinophils | <= 0.55 * 10^3/µL , > 0.55 * 10^3/µL |
| Alcalic phosphatase | < 36 U/L , 36 – 150 U/L , > 150 U/L |
| Alanine Aminotransferase | <= 35 * U/L , > 35 * U/L |
| Bilirubin (total) | < 0.3 mg/dL , 0.3-1.2 mg/dL , > 1.2 mg/dL |

**Supplementary Table 4 - Logistic regression for analysis of probability of irAE for model 1 including baseline data (Eligible Safety Set).** The p-value of the Chi-Square-test is given. For odds ratios (OR), Wald confidence limits (CL) are given. ICI: Immune checkpoint inhibitor, ECOG: Eastern Cooperative Oncology Group Performance Status, SCC: Squamous Cell Carcinoma, irAE: immune related Adverse Events, WBC: White Blood Cell Count. SOC: System Organ Class, G: Group

| **Subgroup** | **Overall p-value** | **Comparison** | **Odds ratio** | **Lower 95%-CL** | **Upper 95%-CL** |  |
| --- | --- | --- | --- | --- | --- | --- |
| Actual Treatment | 0.0087 | ICI dual vs ICI mono | 1.638 | 1.121 | 2.394 | Different from OR=1 |
|  |  | ICI dual vs ICI other | 1.243 | 0.737 | 2.096 |  |
|  |  | ICI dual vs Non-ICI | 5.224 | 1.489 | 18.330 | Different from OR=1 |
|  |  | ICI mono vs ICI other | 0.759 | 0.434 | 1.327 |  |
|  |  | ICI mono vs Non-ICI | 3.189 | 0.902 | 11.274 |  |
|  |  | ICI other vs Non-ICI | 4.203 | 1.131 | 15.622 | Different from OR=1 |
| Neut-Lym Ratio Baseline Group | 0.1649 | G1+2: <= 3 vs G3: >3 | 1.296 | 0.899 | 1.868 |  |
| BMI Group | 0.1130 | 25-30 vs <25 | 0.986 | 0.675 | 1.441 |  |
|  |  | 25-30 vs >30 | 0.630 | 0.390 | 1.018 |  |
|  |  | <25 vs >30 | 0.639 | 0.406 | 1.007 |  |
| ECOG at Baseline Group | 0.1280 | 0 vs 1+ | 1.320 | 0.923 | 1.889 |  |
| Liver Metastasis at Baseline | 0.0129 | No vs Yes | 1.761 | 1.128 | 2.751 | Different from OR=1 |
| Pre-therapy | 0.0292 | Both vs Chemotherapy | 1.367 | 0.875 | 2.137 |  |
|  |  | Both vs Neither | 0.910 | 0.573 | 1.445 |  |
|  |  | Both vs Radiotherapy | 3.553 | 1.152 | 10.958 | Different from OR=1 |
|  |  | Chemotherapy vs Neither | 0.665 | 0.450 | 0.984 | Different from OR=1 |
|  |  | Chemotherapy vs Radiotherapy | 2.599 | 0.853 | 7.921 |  |
|  |  | Neither vs Radiotherapy | 3.905 | 1.272 | 11.991 | Different from OR=1 |
| WBC Baseline Group | 0.0533 | G1: < 4.5 *10/µL vs G2: 4.5 - 11 *10/µL | 0.828 | 0.445 | 1.539 |  |
|  |  | G1: < 4.5 *10/µL vs G3: > 11 *10/µL | 1.638 | 0.731 | 3.672 |  |
|  |  | G2: 4.5 - 11 *10/µL vs G3: > 11 *10/µL | 1.979 | 1.129 | 3.471 | Different from OR=1 |

**Supplementary Table 5 - Logistic regression for analysis of probability of irAE for model 2 including all data (Eligible Safety Set).** The p-value of the Chi-Square-test is given. For odds ratios (OR), Wald confidence limits (CL) are given. ICI: Immune checkpoint inhibitor, ECOG: Eastern Cooperative Oncology Group Performance Status, SCC: Squamous Cell Carcinoma, irAE: immune related Adverse Events, WBC: White Blood Cell Count. SOC: System Organ Class, G: Group

| **Subgroup** | **Overall p-value** | **Comparison** | **Odds ratio** | **Lower 95%-CL** | **Upper 95%-CL** |  |
| --- | --- | --- | --- | --- | --- | --- |
| Actual Treatment | 0.0061 | ICI dual vs ICI mono | 1.687 | 1.137 | 2.503 | Different from OR=1 |
|  |  | ICI dual vs ICI other | 1.197 | 0.695 | 2.059 |  |
|  |  | ICI dual vs Non-ICI | 5.702 | 1.588 | 20.474 | Different from OR=1 |
|  |  | ICI mono vs ICI other | 0.710 | 0.395 | 1.274 |  |
|  |  | ICI mono vs Non-ICI | 3.380 | 0.929 | 12.298 |  |
|  |  | ICI other vs Non-ICI | 4.764 | 1.242 | 18.280 | Different from OR=1 |
| Concomitant Antibiotics Group | 0.0018 | No vs Yes | 0.561 | 0.390 | 0.807 | Different from OR=1 |
| Neutrophils Baseline Group | 0.0449 | G1: < 2.6 *10/µL vs G2: 2.6 - 8.5 *10/µL | 0.963 | 0.435 | 2.132 |  |
|  |  | G1: < 2.6 *10/µL vs G3: > 8.5 *10/µL | 2.064 | 0.789 | 5.402 |  |
|  |  | G2: 2.6 - 8.5 *10/µL vs G3: > 8.5 *10/µL | 2.145 | 1.176 | 3.910 | Different from OR=1 |
| BMI Group | 0.0390 | 25-30 vs <25 | 0.835 | 0.561 | 1.242 |  |
|  |  | 25-30 vs >30 | 0.523 | 0.316 | 0.865 | Different from OR=1 |
|  |  | <25 vs >30 | 0.626 | 0.391 | 1.002 |  |
| Treatment Duration Group | 0.0008 | < median vs >= median | 0.548 | 0.385 | 0.779 | Different from OR=1 |
| Liver Metastasis at Baseline | 0.0971 | No vs Yes | 1.485 | 0.931 | 2.369 |  |
| Pre-therapy | 0.0230 | Both vs Chemotherapy | 1.353 | 0.863 | 2.123 |  |
|  |  | Both vs Neither | 0.935 | 0.584 | 1.500 |  |
|  |  | Both vs Radiotherapy | 4.433 | 1.397 | 14.069 | Different from OR=1 |
|  |  | Chemotherapy vs Neither | 0.691 | 0.459 | 1.041 |  |
|  |  | Chemotherapy vs Radiotherapy | 3.276 | 1.058 | 10.142 | Different from OR=1 |
|  |  | Neither vs Radiotherapy | 4.739 | 1.518 | 14.791 | Different from OR=1 |
| Concomitant Steroids Group | 0.0005 | No vs Yes | 0.522 | 0.362 | 0.753 | Different from OR=1 |
| Previous Antibiotics Group | 0.1721 | No vs Yes | 1.736 | 0.787 | 3.830 |  |

**Supplementary Table 6 - Logistic regression for analysis of probability of serious IRAEs for model 1 including baseline data (Eligible Safety Set).** The p-value of the Chi-Square-test is given. For odds ratios, Wald confidence limits (CL) are given. ICI: Immune checkpoint inhibitor, ECOG: Eastern Cooperative Oncology Group Performance Status, SCC: Squamous Cell Carcinoma, irAE: immune related Adverse Events, WBC: White Blood Cell Count. SOC: System Organ Class, G: Group

| **Subgroup** | **Overall p-value** | **Comparison** | **Odds ratio** | **Lower 95%-CL** | **Upper 95%-CL** |  |
| --- | --- | --- | --- | --- | --- | --- |
| Actual Treatment | <.0001 | ICI dual vs ICI mono | 3.400 | 1.874 | 6.170 | Different from OR=1 |
|  |  | ICI dual vs ICI other | 6.150 | 2.165 | 17.469 | Different from OR=1 |
|  |  | ICI dual vs Non-ICI | 0.659 | 0.040 | 10.884 |  |
|  |  | ICI mono vs ICI other | 1.809 | 0.580 | 5.641 |  |
|  |  | ICI mono vs Non-ICI | 0.194 | 0.011 | 3.344 |  |
|  |  | ICI other vs Non-ICI | 0.107 | 0.005 | 2.125 |  |
| Cancer Type | 0.0047 | SCC vs non-SCC | 0.057 | 0.008 | 0.414 | Different from OR=1 |
| Liver Metastasis at Baseline | 0.0322 | No vs Yes | 2.235 | 1.071 | 4.667 | Different from OR=1 |

**Supplementary Table 7 - Logistic regression for analysis of probability of serious IRAEs for model 2 including all data (Eligible Safety Set).** The p-value of the Chi-Square-test is given. For odds ratios (OR), Wald confidence limits (CL) are given. ICI: Immune checkpoint inhibitor, ECOG: Eastern Cooperative Oncology Group Performance Status, SCC: Squamous Cell Carcinoma, irAE: immune related Adverse Events, WBC: White Blood Cell Count. SOC: System Organ Class, G: Group

| **Subgroup** | **Overall p-value** | **Comparison** | **Odds ratio** | **Lower 95%-CL** | **Upper 95%-CL** |  |
| --- | --- | --- | --- | --- | --- | --- |
| Actual Treatment | 0.0002 | ICI dual vs ICI mono | 3.102 | 1.679 | 5.731 | Different from OR=1 |
|  |  | ICI dual vs ICI other | 5.305 | 1.835 | 15.336 | Different from OR=1 |
|  |  | ICI dual vs Non-ICI | 0.648 | 0.039 | 10.880 |  |
|  |  | ICI mono vs ICI other | 1.710 | 0.539 | 5.423 |  |
|  |  | ICI mono vs Non-ICI | 0.209 | 0.012 | 3.681 |  |
|  |  | ICI other vs Non-ICI | 0.122 | 0.006 | 2.475 |  |
| Cancer Type | 0.0140 | SCC vs non-SCC | 0.081 | 0.011 | 0.601 | Different from OR=1 |
| Liver Metastasis at Baseline | 0.0489 | No vs Yes | 2.140 | 1.004 | 4.563 | Different from OR=1 |
| Concomitant Steroids Group | <.0001 | No vs Yes | 0.280 | 0.151 | 0.521 | Different from OR=1 |
| Concomitant Antibiotics Group | 0.0305 | No vs Yes | 0.572 | 0.345 | 0.949 | Different from OR=1 |

**Supplementary Table 8 - Cox regression for analysis of progression-free survival (Eligible Safety set).** The p-value of the Chi-Square-test is given. For Hazard ratios, Wald confidence limits (CL) are given. ICI: Immune checkpoint inhibitor, ECOG: Eastern Cooperative Oncology Group Performance Status, SCC: Squamous Cell Carcinoma, irAE: immune related Adverse Events, WBC: White Blood Cell Count. SOC: System Organ Class, G: Group

| **Subgroup** | **Overall p-value** | **Comparison** | **Hazard ratio** | **Lower 95%-CL** | **Upper 95%-CL** |  |
| --- | --- | --- | --- | --- | --- | --- |
| **Model 1 Baseline Data** | | | | | | |
| Cancer Type | 0.0312 | SCC vs non-SCC | 1.363 | 1.028 | 1.806 | Different from HR=1 |
| LDH Baseline Group | 0.0834 | G1+2: <= 160 U/L vs G3: > 160 U/L | 0.779 | 0.587 | 1.034 |  |
| WBC Baseline Group | 0.0650 | G1: < 4.5 *10/µL vs G2: 4.5 - 11 *10/µL | 1.293 | 0.917 | 1.824 |  |
|  |  | G1: < 4.5 *10/µL vs G3: > 11 *10/µL | 0.984 | 0.652 | 1.484 |  |
|  |  | G2: 4.5 - 11 *10/µL vs G3: > 11 *10/µL | 0.761 | 0.581 | 0.996 | Different from HR=1 |
| Neut-Lym Ratio Baseline Group | <.0001 | G1+2: <= 3 vs G3: >3 | 0.558 | 0.445 | 0.699 | Different from HR=1 |
| Actual Treatment | 0.0415 | ICI dual vs ICI mono | 1.112 | 0.892 | 1.387 |  |
|  |  | ICI dual vs ICI other | 0.730 | 0.550 | 0.967 | Different from HR=1 |
|  |  | ICI dual vs Non-ICI | 0.846 | 0.520 | 1.377 |  |
|  |  | ICI mono vs ICI other | 0.656 | 0.485 | 0.887 | Different from HR=1 |
|  |  | ICI mono vs Non-ICI | 0.761 | 0.465 | 1.246 |  |
|  |  | ICI other vs Non-ICI | 1.160 | 0.677 | 1.988 |  |
| ECOG at Baseline Group | 0.0009 | 0 vs 1+ | 0.715 | 0.586 | 0.872 | Different from HR=1 |
| Treatment Line | <.0001 | First vs Second | 0.655 | 0.531 | 0.807 | Different from HR=1 |
|  |  | First vs Third or more | 0.468 | 0.316 | 0.694 | Different from HR=1 |
|  |  | Second vs Third or more | 0.715 | 0.492 | 1.039 |  |
| Liver Metastasis at Baseline | <.0001 | No vs Yes | 0.522 | 0.418 | 0.652 | Different from HR=1 |
| Previous Antibiotics Group | 0.0090 | No vs Yes | 0.633 | 0.449 | 0.892 | Different from HR=1 |
| **Model 2 All Data** | | | | | | |
| At Least One Serious irAE | 0.0378 | No vs Yes | 1.488 | 1.023 | 2.165 | Different from HR=1 |
| irAE of SOC Gastrointestinal Disorders | 0.0771 | No vs Yes | 1.437 | 0.961 | 2.147 |  |
| Cancer Type | 0.1664 | SCC vs non-SCC | 1.261 | 0.908 | 1.750 |  |
| Actual Treatment | 0.0855 | ICI dual vs ICI mono | 1.051 | 0.833 | 1.327 |  |
|  |  | ICI dual vs ICI other | 0.751 | 0.557 | 1.012 |  |
|  |  | ICI dual vs Non-ICI | 0.714 | 0.434 | 1.176 |  |
|  |  | ICI mono vs ICI other | 0.715 | 0.525 | 0.972 | Different from HR=1 |
|  |  | ICI mono vs Non-ICI | 0.680 | 0.405 | 1.141 |  |
|  |  | ICI other vs Non-ICI | 0.951 | 0.544 | 1.663 |  |
| ECOG at Baseline Group | 0.1716 | 0 vs 1+ | 0.868 | 0.708 | 1.064 |  |
| Treatment Duration Group | <.0001 | < median vs >= median | 6.924 | 5.468 | 8.767 | Different from HR=1 |
| Treatment Line | 0.0004 | First vs Second | 3.441 | 1.509 | 7.849 | Different from HR=1 |
|  |  | First vs Third or more | 2.093 | 0.850 | 5.157 |  |
|  |  | Second vs Third or more | 0.608 | 0.420 | 0.881 | Different from HR=1 |
| Metastasis at Baseline | 0.1815 | No vs Yes | 1.268 | 0.895 | 1.796 |  |
|  | 0.1815 | No vs Yes | 1.268 | 0.895 | 1.796 |  |
| Liver Metastasis at Baseline | <.0001 | No vs Yes | 0.520 | 0.413 | 0.655 | Different from HR=1 |
| Pre-therapy | 0.0050 | Both vs Chemotherapy | 1.020 | 0.783 | 1.330 |  |
|  |  | Both vs Neither | 4.363 | 1.875 | 10.148 | Different from HR=1 |
|  |  | Both vs Radiotherapy | 3.278 | 1.288 | 8.345 | Different from HR=1 |
|  |  | Chemotherapy vs Neither | 4.275 | 1.851 | 9.876 | Different from HR=1 |
|  |  | Chemotherapy vs Radiotherapy | 3.213 | 1.251 | 8.250 | Different from HR=1 |
|  |  | Neither vs Radiotherapy | 0.751 | 0.466 | 1.211 |  |
| Concomitant Steroids Group | 0.0170 | No vs Yes | 1.281 | 1.045 | 1.570 | Different from HR=1 |
| WBC Baseline Group | 0.0387 | G1: < 4.5 *10/µL vs G2: 4.5 - 11 *10/µL | 0.910 | 0.585 | 1.414 |  |
|  |  | G1: < 4.5 *10/µL vs G3: > 11 *10/µL | 0.489 | 0.256 | 0.936 | Different from HR=1 |
|  |  | G2: 4.5 - 11 *10/µL vs G3: > 11 *10/µL | 0.538 | 0.331 | 0.873 | Different from HR=1 |
| Neutrophils Baseline Group | 0.1371 | G1: < 2.6 *10/µL vs G2: 2.6 - 8.5 *10/µL | 1.608 | 0.911 | 2.839 |  |
|  |  | G1: < 2.6 *10/µL vs G3: > 8.5 *10/µL | 2.171 | 1.008 | 4.676 | Different from HR=1 |
|  |  | G2: 2.6 - 8.5 *10/µL vs G3: > 8.5 *10/µL | 1.350 | 0.814 | 2.240 |  |
| Neut-Lym Ratio Baseline Group | <.0001 | G1+2: <= 3 vs G3: >3 | 0.604 | 0.476 | 0.766 | Different from HR=1 |

**Supplementary Table 9 - Cox regression for analysis for duration of response for model 1 based on baseline data (Eligible Safety set).** The p-value of the Chi-Square-test is given. For Hazard ratios, Wald confidence limits (CL) are given. ICI: Immune checkpoint inhibitor, ECOG: Eastern Cooperative Oncology Group Performance Status, SCC: Squamous Cell Carcinoma, irAE: immune related Adverse Events, WBC: White Blood Cell Count. SOC: System Organ Class, G: Group

| **Subgroup** | **Overall p-value** | **Comparison** | **Hazard ratio** | **Lower 95%-CL** | **Upper 95%-CL** |  |
| --- | --- | --- | --- | --- | --- | --- |
| Cancer Type | 0.0843 | SCC vs non-SCC | 2.070 | 0.906 | 4.728 |  |
| Total Bilirubin Baseline Group | 0.0940 | G1: < 0.3 mg/dL vs G2+3: >= 0.3 mg/dL | 0.612 | 0.344 | 1.087 |  |
| Actual Treatment | 0.0004 | ICI dual vs ICI mono | 2.728 | 1.554 | 4.789 | Different from HR=1 |
|  |  | ICI dual vs ICI other | 0.657 | 0.363 | 1.188 |  |
|  |  | ICI dual vs Non-ICI | 0.878 | 0.206 | 3.749 |  |
|  |  | ICI mono vs ICI other | 0.241 | 0.118 | 0.490 | Different from HR=1 |
|  |  | ICI mono vs Non-ICI | 0.322 | 0.075 | 1.382 |  |
|  |  | ICI other vs Non-ICI | 1.337 | 0.283 | 6.319 |  |
| Metastasis at Baseline | 0.0290 | No vs Yes | 2.316 | 1.090 | 4.921 | Different from HR=1 |
| Liver Metastasis at Baseline | 0.1045 | No vs Yes | 0.636 | 0.368 | 1.098 |  |
| Previous Antibiotics Group | <.0001 | No vs Yes | 0.184 | 0.083 | 0.410 | Different from HR=1 |
| LDH Baseline Group | 0.0377 | G1+2: <= 160 U/L vs G3: > 160 U/L | 0.488 | 0.248 | 0.960 | Different from HR=1 |

**Supplementary Table 10 - Cox regression for analysis for duration of response for model 2 based on all data (Eligible Safety set).** The p-value of the Chi-Square-test is given. For Hazard ratios, Wald confidence limits (CL) are given. ICI: Immune checkpoint inhibitor, ECOG: Eastern Cooperative Oncology Group Performance Status, SCC: Squamous Cell Carcinoma, irAE: immune related Adverse Events, WBC: White Blood Cell Count. SOC: System Organ Class, G: Group

| **Subgroup** | **Overall p-value** | **Comparison** | **Hazard ratio** | **Lower 95%-CL** | **Upper 95%-CL** |  |
| --- | --- | --- | --- | --- | --- | --- |
| Cancer Type | 0.0891 | SCC vs non-SCC | 2.206 | 0.886 | 5.490 |  |
| Actual Treatment | <.0001 | ICI dual vs ICI mono | 2.940 | 1.652 | 5.233 | Different from HR=1 |
|  |  | ICI dual vs ICI other | 0.595 | 0.324 | 1.092 |  |
|  |  | ICI dual vs Non-ICI | 0.776 | 0.179 | 3.371 |  |
|  |  | ICI mono vs ICI other | 0.202 | 0.098 | 0.417 | Different from HR=1 |
|  |  | ICI mono vs Non-ICI | 0.264 | 0.059 | 1.188 |  |
|  |  | ICI other vs Non-ICI | 1.304 | 0.264 | 6.434 |  |
| Treatment Duration Group | <.0001 | < median vs >= median | 3.702 | 1.978 | 6.928 | Different from HR=1 |
| Metastasis at Baseline | 0.0857 | No vs Yes | 2.010 | 0.907 | 4.455 |  |
| Liver Metastasis at Baseline | 0.0098 | No vs Yes | 0.470 | 0.265 | 0.834 | Different from HR=1 |
| Concomitant Steroids Group | 0.0128 | No vs Yes | 1.832 | 1.138 | 2.951 | Different from HR=1 |
| Previous Antibiotics Group | 0.0031 | No vs Yes | 0.305 | 0.139 | 0.669 | Different from HR=1 |
| LDH Baseline Group | 0.0174 | G1+2: <= 160 U/L vs G3: > 160 U/L | 0.428 | 0.213 | 0.861 | Different from HR=1 |

**Supplementary Table 11 - Cox regression for analysis of treatment-free survival for model 1 based on baseline data (Eligible Safety set).** The p-value of the Chi-Square-test is given. For Hazard ratios, Wald confidence limits (CL) are given. ICI: Immune checkpoint inhibitor, ECOG: Eastern Cooperative Oncology Group Performance Status, SCC: Squamous Cell Carcinoma, irAE: immune related Adverse Events, WBC: White Blood Cell Count. SOC: System Organ Class, G: Group

| **Subgroup** | **Overall p-value** | **Comparison** | **Hazard ratio** | **Lower 95%-CL** | **Upper 95%-CL** |  |
| --- | --- | --- | --- | --- | --- | --- |
| Lymphocytes Baseline Group | 0.0367 | G1: < 0.77 *10/µL vs G2+3: >= 0.77 *10/µL | 1.463 | 1.024 | 2.092 | Different from HR=1 |
| Neutrophils Baseline Group | 0.1112 | G1: < 2.6 *10/µL vs G2: 2.6 - 8.5 *10/µL | 1.195 | 0.547 | 2.611 |  |
|  |  | G1: < 2.6 *10/µL vs G3: > 8.5 *10/µL | 0.777 | 0.326 | 1.852 |  |
|  |  | G2: 2.6 - 8.5 *10/µL vs G3: > 8.5 *10/µL | 0.650 | 0.432 | 0.979 | Different from HR=1 |
| Neut-Lym Ratio Baseline Group | 0.0009 | G1+2: <= 3 vs G3: >3 | 0.517 | 0.350 | 0.763 | Different from HR=1 |
| ECOG at Baseline Group | 0.0546 | 0 vs 1+ | 0.744 | 0.550 | 1.006 |  |
| Treatment Line | 0.0041 | First vs Second | 0.571 | 0.407 | 0.802 | Different from HR=1 |
|  |  | First vs Third or more | 0.807 | 0.439 | 1.486 |  |
|  |  | Second vs Third or more | 1.414 | 0.806 | 2.480 |  |
| Liver Metastasis at Baseline | <.0001 | No vs Yes | 0.430 | 0.309 | 0.598 | Different from HR=1 |
|  |  |  |  |  |  |  |

**Supplementary Table 12 - Cox regression for analysis of treatment-free survival for model 2 based on all data (Eligible Safety set).** The p-value of the Chi-Square-test is given. For Hazard ratios, Wald confidence limits (CL) are given. ICI: Immune checkpoint inhibitor, ECOG: Eastern Cooperative Oncology Group Performance Status, SCC: Squamous Cell Carcinoma, irAE: immune related Adverse Events, WBC: White Blood Cell Count. SOC: System Organ Class, G: Group

| **Subgroup** | **Overall p-value** | **Comparison** | **Hazard ratio** | **Lower 95%-CL** | **Upper 95%-CL** |  |
| --- | --- | --- | --- | --- | --- | --- |
| Rarity of Rarest irAE | 0.1096 | Rare (< 1%) vs Uncommon (1-10%) | 0.585 | 0.303 | 1.128 |  |
| Age Group | 0.1514 | -64 vs 65-74 | 0.710 | 0.499 | 1.010 |  |
|  |  | -64 vs 75+ | 0.754 | 0.504 | 1.130 |  |
|  |  | 65-74 vs 75+ | 1.063 | 0.740 | 1.527 |  |
| BMI Group | 0.0588 | 25-30 vs <25 | 0.762 | 0.541 | 1.074 |  |
|  |  | 25-30 vs >30 | 1.230 | 0.781 | 1.937 |  |
|  |  | <25 vs >30 | 1.614 | 1.054 | 2.470 | Different from HR=1 |
| ECOG at Baseline Group | 0.1818 | 0 vs 1+ | 0.799 | 0.574 | 1.111 |  |
| Treatment Duration Group | <.0001 | < median vs >= median | 3.143 | 2.227 | 4.437 | Different from HR=1 |
| Treatment Line | 0.0181 | First vs Second | 3.039 | 0.742 | 12.452 |  |
|  |  | First vs Third or more | 6.279 | 1.349 | 29.224 | Different from HR=1 |
|  |  | Second vs Third or more | 2.066 | 1.141 | 3.740 | Different from HR=1 |
| Metastasis at Baseline | 0.0050 | No vs Yes | 1.930 | 1.220 | 3.053 | Different from HR=1 |
| Liver Metastasis at Baseline | <.0001 | No vs Yes | 0.504 | 0.358 | 0.708 | Different from HR=1 |
| Pre-therapy | 0.1086 | Both vs Chemotherapy | 0.744 | 0.521 | 1.063 |  |
|  |  | Both vs Neither | 2.893 | 0.694 | 12.059 |  |
|  |  | Both vs Radiotherapy | 3.793 | 0.779 | 18.481 |  |
|  |  | Chemotherapy vs Neither | 3.889 | 0.946 | 15.994 |  |

**Supplementary Table 13 - Logistic regression for analysis of objective response rate for model 1 based on baseline data (Eligible Safety set).** The p-value of the Chi-Square-test is given. For Hazard ratios, Wald confidence limits (CL) are given. ICI: Immune checkpoint inhibitor, ECOG: Eastern Cooperative Oncology Group Performance Status, SCC: Squamous Cell Carcinoma, irAE: immune related Adverse Events, WBC: White Blood Cell Count. SOC: System Organ Class, G: Group

| **Subgroup** | **Overall p-value** | **Comparison** | **Hazard ratio** | **Lower 95%-CL** | **Upper 95%-CL** |  |
| --- | --- | --- | --- | --- | --- | --- |
| Cancer Type | 0.0494 | SCC vs non-SCC | 0.494 | 0.245 | 0.998 | Different from OR=1 |
| WBC Baseline Group | 0.0246 | G1: < 4.5 *10/µL vs G2: 4.5 - 11 *10/µL | 0.521 | 0.259 | 1.050 |  |
|  |  | G1: < 4.5 *10/µL vs G3: > 11 *10/µL | 1.005 | 0.413 | 2.448 |  |
|  |  | G2: 4.5 - 11 *10/µL vs G3: > 11 *10/µL | 1.929 | 1.058 | 3.517 | Different from OR=1 |
| Neut-Lym Ratio Baseline Group | <.0001 | G1+2: <= 3 vs G3: >3 | 2.278 | 1.558 | 3.329 | Different from OR=1 |
| Actual Treatment | 0.1669 | ICI dual vs ICI mono | 0.653 | 0.439 | 0.972 | Different from OR=1 |
|  |  | ICI dual vs ICI other | 0.816 | 0.458 | 1.454 |  |
|  |  | ICI dual vs Non-ICI | 1.485 | 0.356 | 6.198 |  |
|  |  | ICI mono vs ICI other | 1.250 | 0.689 | 2.270 |  |
|  |  | ICI mono vs Non-ICI | 2.274 | 0.541 | 9.555 |  |
|  |  | ICI other vs Non-ICI | 1.819 | 0.400 | 8.262 |  |
| ECOG at Baseline Group | <.0001 | 0 vs 1+ | 2.230 | 1.513 | 3.285 | Different from OR=1 |
| Treatment Line | 0.0015 | First vs Second | 1.949 | 1.342 | 2.832 | Different from OR=1 |
|  |  | First vs Third or more | 2.251 | 0.871 | 5.817 |  |
|  |  | Second vs Third or more | 1.155 | 0.454 | 2.940 |  |
| Liver Metastasis at Baseline | 0.0093 | No vs Yes | 1.894 | 1.170 | 3.065 | Different from OR=1 |

**Supplementary Table 14 - Logistic regression for analysis of objective response rate for model 2 based on all data (Eligible Safety set).** The p-value of the Chi-Square-test is given. For Hazard ratios, Wald confidence limits (CL) are given. ICI: Immune checkpoint inhibitor, ECOG: Eastern Cooperative Oncology Group Performance Status, SCC: Squamous Cell Carcinoma, irAE: immune related Adverse Events, WBC: White Blood Cell Count. SOC: System Organ Class, G: Group

| **Subgroup** | **Overall p-value** | **Comparison** | **Hazard ratio** | **Lower 95%-CL** | **Upper 95%-CL** |  |
| --- | --- | --- | --- | --- | --- | --- |
| Actual Treatment | 0.0693 | ICI dual vs ICI mono | 0.547 | 0.338 | 0.886 | Different from OR=1 |
|  |  | ICI dual vs ICI other | 0.929 | 0.471 | 1.835 |  |
|  |  | ICI dual vs Non-ICI | 1.506 | 0.320 | 7.094 |  |
|  |  | ICI mono vs ICI other | 1.697 | 0.825 | 3.491 |  |
|  |  | ICI mono vs Non-ICI | 2.751 | 0.586 | 12.917 |  |
|  |  | ICI other vs Non-ICI | 1.621 | 0.305 | 8.616 |  |
| ECOG at Baseline Group | 0.0305 | 0 vs 1+ | 1.653 | 1.049 | 2.606 | Different from OR=1 |
| Treatment Duration Group | <.0001 | < median vs >= median | 0.079 | 0.048 | 0.129 | Different from OR=1 |
| Liver Metastasis at Baseline | 0.1063 | No vs Yes | 1.595 | 0.905 | 2.812 |  |
| Pre-therapy | 0.0443 | Both vs Chemotherapy | 0.666 | 0.349 | 1.273 |  |
|  |  | Both vs Neither | 0.413 | 0.214 | 0.797 | Different from OR=1 |
|  |  | Both vs Radiotherapy | 0.523 | 0.188 | 1.455 |  |
|  |  | Chemotherapy vs Neither | 0.619 | 0.380 | 1.011 |  |
|  |  | Chemotherapy vs Radiotherapy | 0.786 | 0.291 | 2.120 |  |
|  |  | Neither vs Radiotherapy | 1.268 | 0.467 | 3.445 |  |
| Concomitant Antibiotics Group | 0.0075 | No vs Yes | 0.558 | 0.364 | 0.855 | Different from OR=1 |
| Neut-Lym Ratio Baseline Group | 0.0059 | G1+2: <= 3 vs G3: >3 | 1.864 | 1.197 | 2.903 | Different from OR=1 |

**Supplementary Table 15 - Logistic regression for analysis of disease control rate for model 1 based on baseline data (Eligible Safety set).** The p-value of the Chi-Square-test is given. For Hazard ratios, Wald confidence limits (CL) are given. ICI: Immune checkpoint inhibitor, ECOG: Eastern Cooperative Oncology Group Performance Status, SCC: Squamous Cell Carcinoma, irAE: immune related Adverse Events, WBC: White Blood Cell Count. SOC: System Organ Class, G: Group

| **Subgroup** | **Overall p-value** | **Comparison** | **Hazard ratio** | **Lower 95%-CL** | **Upper 95%-CL** |  |
| --- | --- | --- | --- | --- | --- | --- |
| Cancer Type | 0.0352 | SCC vs non-SCC | 0.235 | 0.061 | 0.905 | Different from OR=1 |
| Neut-Lym Ratio Baseline Group | 0.0081 | G1+2: <= 3 vs G3: >3 | 1.954 | 1.190 | 3.207 | Different from OR=1 |
| Total Bilirubin Baseline Group | 0.0849 | G1: < 0.3 mg/dL vs G2+3: >= 0.3 mg/dL | 1.555 | 0.941 | 2.568 |  |
| Actual Treatment | <.0001 | ICI dual vs ICI mono | 0.255 | 0.152 | 0.428 | Different from OR=1 |
|  |  | ICI dual vs ICI other | 1.542 | 0.563 | 4.218 |  |
|  |  | ICI dual vs Non-ICI | 0.187 | 0.035 | 1.006 |  |
|  |  | ICI mono vs ICI other | 6.041 | 2.228 | 16.383 | Different from OR=1 |
|  |  | ICI mono vs Non-ICI | 0.732 | 0.141 | 3.800 |  |
|  |  | ICI other vs Non-ICI | 0.121 | 0.018 | 0.813 | Different from OR=1 |
| ECOG at Baseline Group | 0.1557 | 0 vs 1+ | 1.467 | 0.864 | 2.490 |  |
| Liver Metastasis at Baseline | 0.0003 | No vs Yes | 5.217 | 2.153 | 12.641 | Different from OR=1 |
| Pre-therapy | 0.0223 | Both vs Chemotherapy | 0.605 | 0.283 | 1.294 |  |
|  |  | Both vs Neither | 0.391 | 0.179 | 0.851 | Different from OR=1 |
|  |  | Both vs Radiotherapy | 0.242 | 0.081 | 0.722 | Different from OR=1 |
|  |  | Chemotherapy vs Neither | 0.646 | 0.379 | 1.102 |  |
|  |  | Chemotherapy vs Radiotherapy | 0.400 | 0.142 | 1.124 |  |
|  |  | Neither vs Radiotherapy | 0.619 | 0.220 | 1.741 |  |
| Previous Antibiotics Group | 0.0651 | No vs Yes | 6.864 | 0.886 | 53.148 |  |

**Supplementary Table 16 - Logistic regression for analysis of disease control rate for model 2 based on all data (Eligible Safety set).** The p-value of the Chi-Square-test is given. For Hazard ratios, Wald confidence limits (CL) are given. ICI: Immune checkpoint inhibitor, ECOG: Eastern Cooperative Oncology Group Performance Status, SCC: Squamous Cell Carcinoma, irAE: immune related Adverse Events, WBC: White Blood Cell Count. SOC: System Organ Class, G: Group

| **Subgroup** | **Overall p-value** | **Comparison** | **Hazard ratio** | **Lower 95%-CL** | **Upper 95%-CL** |  |
| --- | --- | --- | --- | --- | --- | --- |
| Liver Metastasis at Baseline | 0.0020 | No vs Yes | 4.233 | 1.697 | 10.556 | Different from OR=1 |
| Pre-therapy | 0.0546 | Both vs Chemotherapy | 0.578 | 0.263 | 1.269 |  |
|  |  | Both vs Neither | 0.431 | 0.193 | 0.961 | Different from OR=1 |
|  |  | Both vs Radiotherapy | 0.240 | 0.077 | 0.749 | Different from OR=1 |
|  |  | Chemotherapy vs Neither | 0.746 | 0.423 | 1.316 |  |
|  |  | Chemotherapy vs Radiotherapy | 0.415 | 0.143 | 1.209 |  |
|  |  | Neither vs Radiotherapy | 0.557 | 0.190 | 1.633 |  |
| Previous Antibiotics Group | 0.0704 | No vs Yes | 6.843 | 0.852 | 54.975 |  |
| Neut-Lym Ratio Baseline Group | 0.0439 | G1+2: <= 3 vs G3: >3 | 1.687 | 1.014 | 2.806 | Different from OR=1 |
| Total Bilirubin Baseline Group | 0.1142 | G1: < 0.3 mg/dL vs G2+3: >= 0.3 mg/dL | 1.537 | 0.902 | 2.620 |  |
| Rarity of Rarest irAE | 0.0961 | No irAE vs Rare (< 1%%) | 0.431 | 0.198 | 0.937 | Different from OR=1 |
|  |  | No irAE vs Uncommon (1-10%%) | 0.942 | 0.532 | 1.668 |  |
|  |  | Rare (< 1%%) vs Uncommon (1-10%%) | 2.186 | 0.959 | 4.979 |  |
| Cancer Type | 0.0066 | SCC vs non-SCC | 0.141 | 0.034 | 0.580 | Different from OR=1 |
| Actual Treatment | <.0001 | ICI dual vs ICI mono | 0.244 | 0.142 | 0.418 | Different from OR=1 |
|  |  | ICI dual vs ICI other | 1.723 | 0.612 | 4.848 |  |
|  |  | ICI dual vs Non-ICI | 0.134 | 0.023 | 0.782 | Different from OR=1 |
|  |  | ICI mono vs ICI other | 7.069 | 2.505 | 19.950 | Different from OR=1 |
|  |  | ICI mono vs Non-ICI | 0.552 | 0.100 | 3.036 |  |
|  |  | ICI other vs Non-ICI | 0.078 | 0.011 | 0.562 | Different from OR=1 |
| Age Group | 0.1675 | -64 vs 65-74 | 1.605 | 0.898 | 2.868 |  |
|  |  | -64 vs 75+ | 0.892 | 0.476 | 1.673 |  |
|  |  | 65-74 vs 75+ | 0.556 | 0.281 | 1.099 |  |
| Treatment Duration Group | <.0001 | < median vs >= median | 0.217 | 0.121 | 0.389 | Different from OR=1 |

## Supplementary Figures

**
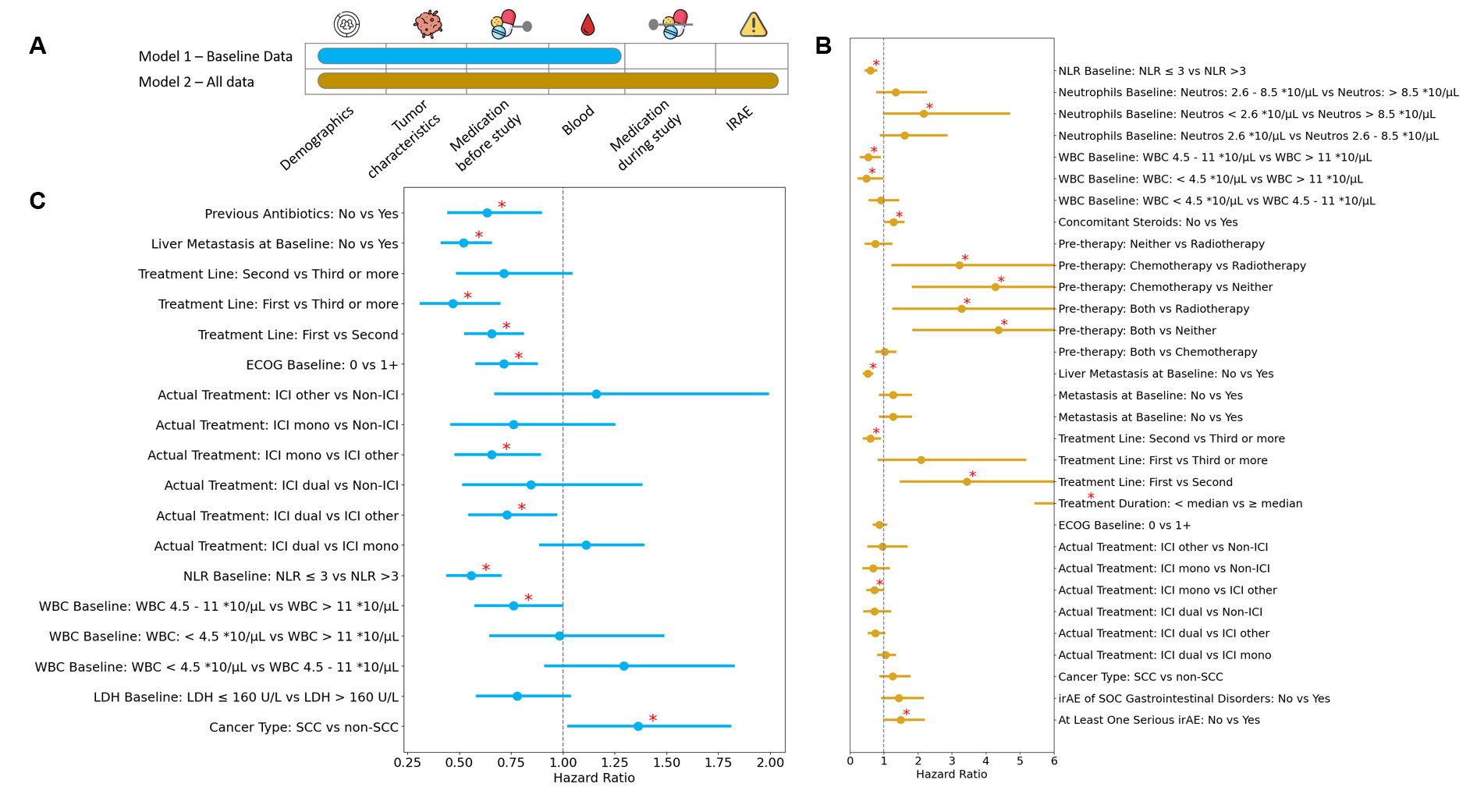
Supplementary Figure 1 - Cox regression for analysis of progression-free survival.** The analyses were performed on the Eligible Safety set. A depicts the grouped variable categories included in the stepwise model building for Model 1, which includes all data available at baseline and Model 2, which includes all variable data. B shows hazard ratios with confidence intervals from variables included in the stepwise modeling process for Model 1, C for Model 2. ICI: Immune checkpoint inhibitor, ECOG: Eastern Cooperative Oncology Group Performance Status, SCC: Squamous Cell Carcinoma, irAE: immune related Adverse Events, WBC: White Blood Cell Count. SOC: System Organ Class, G: Group


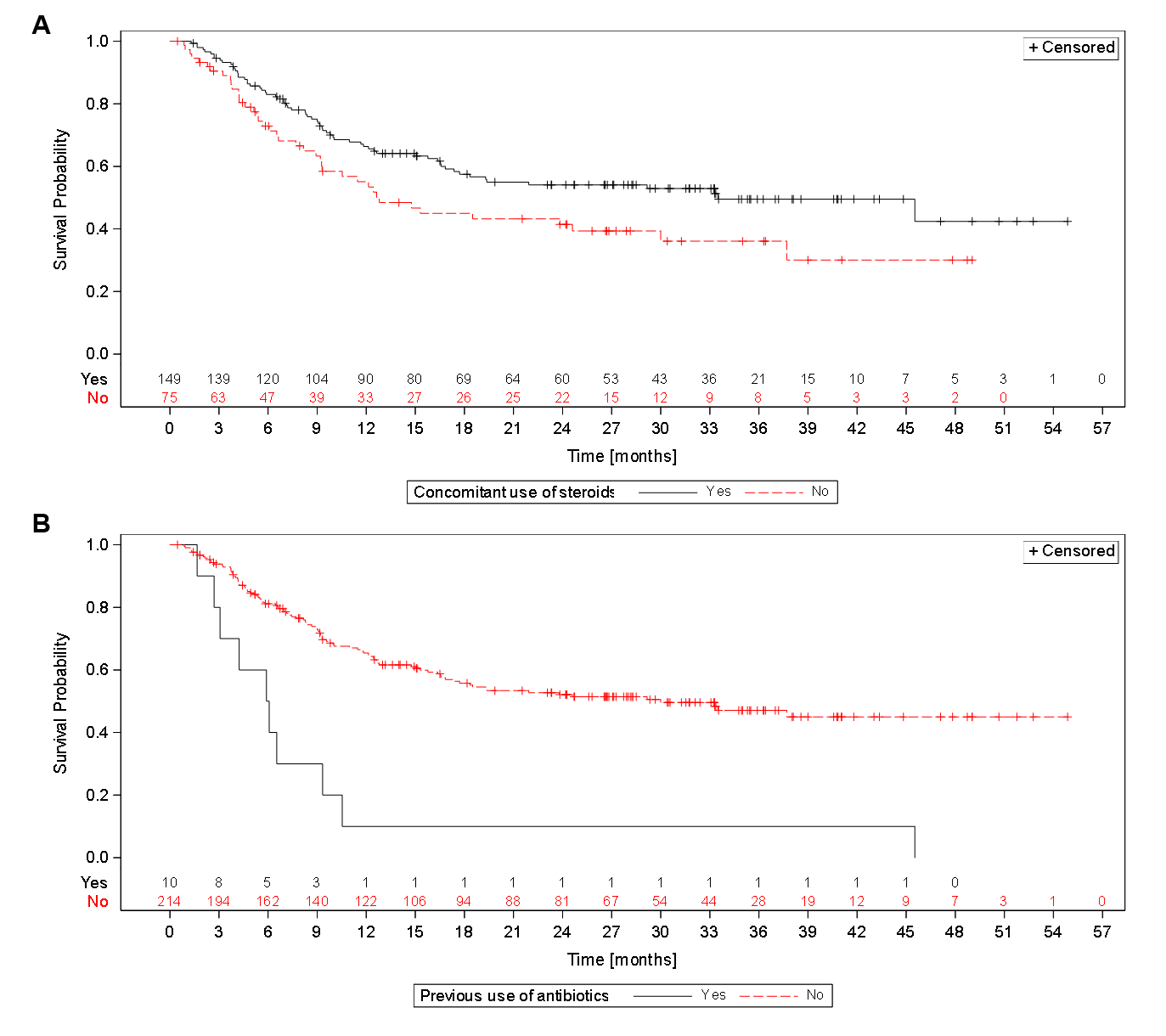


**Supplementary Figure 2 - Kaplan-Meier plots for Duration of Response** A depicts the duration of response for patients who received steroids during study treatment (Yes, in black) vs. those who did not (No, in red). B shows the plot depicting the duration of response for patients who received antibiotics prior to study treatment (Yes, black) and those who did not (No, red).


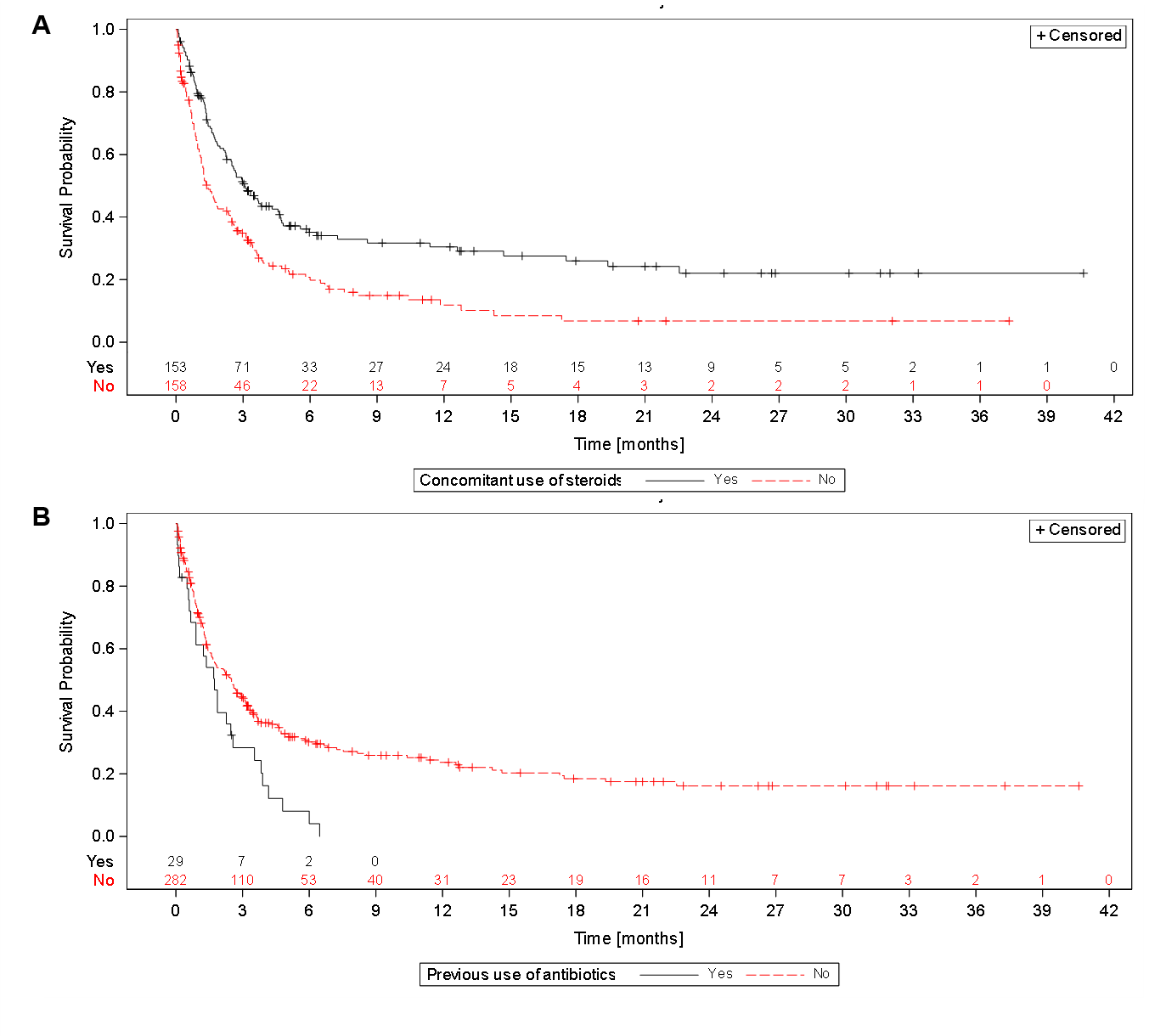
**Supplementary Figure 3 - Kaplan-Meier plots for Treatment Free Survival** (TFS) A depicts the TFS for patients who received steroids during study treatment (Yes, in black) vs. those who did not (No, in red). B shows the plot depicting TFS for patients who received antibiotics prior to study treatment (Yes, black) and those who did not (No, red).
